# Relationship between Sponsorship and Failure Rate of Dental Implants: A Systematic Approach

**DOI:** 10.1371/journal.pone.0010274

**Published:** 2010-04-21

**Authors:** Antoine Popelut, Fabien Valet, Olivier Fromentin, Aurélie Thomas, Philippe Bouchard

**Affiliations:** 1 Department of Periodontology, Service of Odontology, Hôtel-Dieu Hospital, AP-HP, Paris 7 - Denis Diderot University, U.F.R. of Odontology, Paris, France; 2 Department of Biostatistics, Institut Curie, Paris, France; 3 Department of Prosthetic Dentistry, Service of Odontology, Hôtel-Dieu Hospital, AP-HP, Paris 7 - Denis Diderot University, U.F.R. of Odontology, Paris, France; 4 Library of Odontology, Paris 7 - Denis Diderot University, U.F.R. of Odontology, Paris, France; University of British Columbia, Canada

## Abstract

**Background:**

The number of dental implant treatments increases annually. Dental implants are manufactured by competing companies. Systematic reviews and meta-analysis have shown a clear association between pharmaceutical industry funding of clinical trials and pro-industry results. So far, the impact of industry sponsorship on the outcomes and conclusions of dental implant clinical trials has never been explored. The aim of the present study was to examine financial sponsorship of dental implant trials, and to evaluate whether research funding sources may affect the annual failure rate.

**Methods and Findings:**

A systematic approach was used to identify systematic reviews published between January 1993 and December 2008 that specifically deal with the length of survival of dental implants. Primary articles were extracted from these reviews. The failure rate of the dental implants included in the trials was calculated. Data on publication year, Impact Factor, prosthetic design, periodontal status reporting, number of dental implants included in the trials, methodological quality of the studies, presence of a statistical advisor, and financial sponsorship were extracted by two independent reviewers (kappa  = 0.90; CI_95%_ [0.77–1.00]). Univariate quasi-Poisson regression models and multivariate analysis were used to identify variables that were significantly associated with failure rates. Five systematic reviews were identified from which 41 analyzable trials were extracted. The mean annual failure rate estimate was 1.09%.(CI_95%_ [0.84–1.42]). The funding source was not reported in 63% of the trials (26/41). Sixty-six percent of the trials were considered as having a risk of bias (27/41). Given study age, both industry associated (OR = 0.21; CI_95%_ [0.12–0.38]) and unknown funding source trials (OR = 0.33; (CI_95%_ [0.21–0.51]) had a lower annual failure rates compared with non-industry associated trials. A conflict of interest statement was disclosed in 2 trials.

**Conclusions:**

When controlling for other factors, the probability of annual failure for industry associated trials is significantly lower compared with non-industry associated trials. This bias may have significant implications on tooth extraction decision making, research on tooth preservation, and governmental health care policies.

## Introduction

Despite the paucity of epidemiological studies on edentulism in many countries, partial and/or complete edentulism data indicate that patients eligible to receive dental prosthesis remain numerous worldwide [1]. It has been shown that tooth replacement improves the quality of life in edentulous patients [2], [3]. Therefore, tooth replacement is still of paramount importance in health care, even if tremendous efforts have been made by dental researchers in cariology and periodontology to treat and prevent the two main causes of edentulism: dental caries and periodontal diseases [4].

Until the discovery of the osseointegrated implants at the end of the seventies [5], traditional removable dentures or tooth-supported fixed dentures (dental bridges) were universally used to replace missing teeth. However, root-form endosseous dental implants – surgically implantable medical devices aiming to support artificial tooth or group of teeth – have tended to replace the traditional approach. The main reason for the widespread indications of dental implants by practitioners should ideally be the low percentage of annual implant loss, as demonstrated by primary studies and recent systematic reviews and meta-analysis [6]–[10]. In addition, biological reasons, i.e. the preservation of the integrity of the teeth bordering the edentulous area, and economical reasons, i.e. the cost-effectiveness approach showing that implant therapy could be more cost-effective than traditional dental bridge therapy [11], [12], may be advocated. From the patient's point of view, dental implants are more popular than removable dentures or dental bridges because they can offer fixed solutions when traditional tooth replacement therapies cannot.

Therefore, osseointegrated implant discovery has completely changed the therapeutic approach of tooth replacement. Today, 3 journals are integrally dedicated to implant dentistry, and one of them is in the top 10 of the 55 impact factor dental journals. Nevertheless, due to the lack of comparative trials, there is still no evidence that dental implant therapy performs better than traditional therapy, and that one implant brand is more effective than another.

The number of dentists placing dental implants is increasing annually. Estimates indicate that the number of dental implant procedures performed in Europe increased to 3,527,000 in 2008, representing 8.1% growth over 2007 [13]. Markets for dental implants have been estimated at $3.4 billion in 2008, and are anticipated to reach $8.1 billion by 2015 [14]. Implant companies are traded on the stock exchange. Dental implants are manufactured by competing companies. In Europe, 4 companies capture close to 60% of the market [13].

Systematic reviews and meta-analysis have shown a clear association between pharmaceutical industry funding of clinical trials and pro-industry results [15], [16]. So far, to our knowledge, the impact of dental industry sponsorship on the outcomes and conclusions of clinical trials has never been explored. As shown above, dental implant therapy appears to be an interesting example when investigating the influence of funding source on study results. A sponsorship bias in oral implant research may have an adverse effect, not only on preventive dental research but also on teeth preservation. Traditional prosthetic approaches such as tooth-supported fixed dentures need to preserve the teeth; whereas, dental implant therapies require edentulous areas, which are the result of tooth loss. Therefore, in light of the high percentage of success of implant therapy, dentists may be incited to extract teeth instead of making efforts to preserve dental organs.

The aim of the present study was to examine financial sponsorship of dental implant articles, and to determine whether research funding source may affect the annual failure rate. We hypothesized that articles exclusively or partially funded by implant companies are more likely to report lower annual failure rates than articles without industry-associated sponsorship.

## Methods

### Study selection

We included only primary articles from systematic reviews that specifically deal with the length of survival of dental implants. The following factors were examined: publication year (available from Medline), Impact Factor, prosthetic design, periodontal status reporting, the number of dental implants included in the studies, methodological quality of the studies, the presence of a statistical advisor, and the financial sponsorship. Study design, sample size, dental implant brand, corresponding author country, and conflict of interest were recorded for descriptive statistics of the sample. More details on the selection of these factors are given below in the “Data extraction” section. We looked for papers published only in English.

### Search strategy

Two independent reviewers (A.P. and O.F.) searched MEDLINE and the Cochrane database of systematic reviews to identify systematic review articles published between January 1993 and December 2008. The MEDLINE search was conducted using the following MeSH terms exploded: “Dental Implants” and “Denture, Partial, Fixed”, in combination with ‘survival’, ‘success’ or ‘complications’ (Table S1). The search was limited to “reviews”, “meta-analysis”, and “humans”. In addition, hand searching was conducted in the following dental journals: *Journal of Periodontology, International Dental Journal, British Dental Journal, Journal of the International Academy of Periodontology, Journal Canadian Dental Association, Swedish Dental Journal, Quintessence International, Journal of Clinical Periodontology, Periodontology 2000, Clinical Oral Implant Research, International Journal of Oral and Maxillofacial Implants, International Journal of Prosthodontic, Journal of Prosthetic Dentistry*.

### Inclusion and exclusion criteria

#### Systematic reviews

Systematic reviews with or without meta-analysis, had to report or to allow for the calculation of the pooled survival rate or the estimated survival rate of dental implants. Implant-supported single crown (IS-SC), implant-supported fixed partial denture (IS-FPD) and implant-tooth supported fixed partial denture (ITS-FPD) were the only prosthetic designs that were included in the present study. Studies included in the articles had to have a mean follow-up of at least 5 years and less than 10 years. We choose a minimum mean follow-up of 5 years because it corresponds to the best scientific evidence in implant dentistry [9]. Our data included different types of dental implants studies because there is no evidence that any specific type of dental implant has superior survival [17].

Fully edentulous patients were excluded from our research. The following dental implants were eliminated from the database because of the suggested increased failure rate: immediate and immediate-delayed dental implants; early and immediate load dental implants; dental implants placed after sinus lift procedures [18]–[20].

#### Primary articles

The same inclusion/exclusion criteria as those used for the systematic review selection was applied for the primary article selection. Data cleaning was applied to the identification of duplicate publications on the same patient cohorts. When the same cohort was analyzed twice at different follow-up times, the article reporting a mean follow-up closest to 5 years was included.

### Data extraction

Data were extracted from the papers included in the final sample. Two independent investigators were involved in the process of extraction (A.P. and O.F.). In the case of disagreement, the two investigators discussed the article and tried to find agreement. When consensus was not reached, a third investigator (P.B.) was involved until agreement was found. The data were extracted on the following publication characteristics:

#### Journal and authors characteristics

For each article, we documented the name of the journal, the year of publication, and the impact factor for the year prior to publication using the Journal of citation reports. When the journal was not indexed, it was assigned a “0” value. The country of origin of the corresponding author was obtained from the article. The conflict of interest statements were also recorded.

#### Study characteristics

The study design of each article was identified as retrospective or prospective. Included studies were submitted to quality assessment according to the following three main criteria: (1) Inclusion/exclusion criteria, (2) Blindness of the examiner, and (3) Drop-out rates. Criteria 1 and 2 were categorized as the following: (1) “Yes”; (2) “No”, when it was specified that the criteria was not used; (3) “Unclear”, when the article did not mentioned the criteria. The third criterion (drop-out rate) was categorized as “Yes” if reported or “Unclear” if not reported. The studies were evaluated as having low risk of bias if at least 2 quality criteria were met; in all other cases the studies were evaluated as having a risk of bias. It was also noted and recorded if an author served as a statistical advisor or if a statistical advisor was indicated in the article. The prosthetic designs were recorded according to three categories: (1) implant-supported single crown (IS-SC), (2) implant-supported fixed partial denture (IS-FPD), and (3) implant-tooth supported fixed partial denture (ITS-FPD). Studies indicating the periodontal status of the included subjects were recorded.

#### Sample characteristics

In each trial, the number of inserted dental implants and the number of implant losses, as well as the mean follow-up, were recorded. Dental implant brands were also identified in each article.

#### Funding source identification

The funding source of each published study was categorized as follows: (1) industry, (2) industry-associated, (3) non-industry, and (4) unknown. The study was categorized as “industry” when it was clearly stated that it was only supported by grants from an implant company, and “industry-associated” when the implant company had a role in the study design (i.e. data collection or analysis, decision to publish, preparation of the manuscript) or in free dental implant providing. The “non-industry” category included studies supported by grants from universities, governmental agencies, independent foundations, and other nonprofits organizations with no industry association. Each institution classified as “non-industry” was carefully verified through Internet searches. When the information was doubtful or not available, the institution was contacted by e-mail. When no information was given on sponsorship, the funding source was categorized as “unknown”.

### Statistical analysis

Collected data were organized into a spreadsheet using a computer program (Excel, Microsoft, Redmond, WA). After proofing for entry errors, the database was locked and loaded in statistical software program by a statistician (F.V.) blind to the study selection and data extraction. The industry and industry-associated funding-source categories were collapsed for the analysis. All statistical analyses were performed with R 2.10.0 software (R Foundation for Statistical Computing, Vienna, Austria) on PC architecture.

Descriptive statistics were reported as numbers and percentages. The Kappa statistic [21] was used to assess interrater reliability between the two independent reviewers (A.P. and O.F.) using the funding source scale.

The total exposure time of each included study selected from systematic reviews was used for the 5-year estimated survival rate calculation. Failure rate was calculated by dividing the number of events (implant loss) in the numerator by the total exposure time in the denominator [9], [10], [22]. Implant loss was extracted from the publications. The total exposure time was calculated by the sum of *i)* exposure time of implants followed for the whole observation period, *ii)* exposure time of the implants lost during the observation period, and *iii)* exposure time of the implants that did not complete the observation period. Exact CI_95%_ for failure rate estimates was calculated using the relationship between Poisson and Chi-square distributions [23]. Five-year survival proportions were calculated via the relationship between event rate and survival function S [S(T)  =  exp(-T x event rate)] by assuming constant event rates [24].

The total number of events was considered to be Poisson distributed for a given sum of implant exposure years. The Poisson regression model, with a logarithmic link-function and a total exposure time per study as an offset variable, was used [25]. We used Breslow's recommendation to detect overdispersion [26], and a quasi-Poisson model for handling heterogeneity [25].

Consequently, univariate quasi-Poisson regression models were used to find variables that were significantly associated with failure rates. A multivariate analysis was then performed to identify the variables significantly associated with failure rates. For model selection, a deviance approach with a “drop-one” algorithm was used [27]. An F-ratio statistic was calculated to compare models with and without each of the discarded variables [28].

## Results

**Figure 1:**
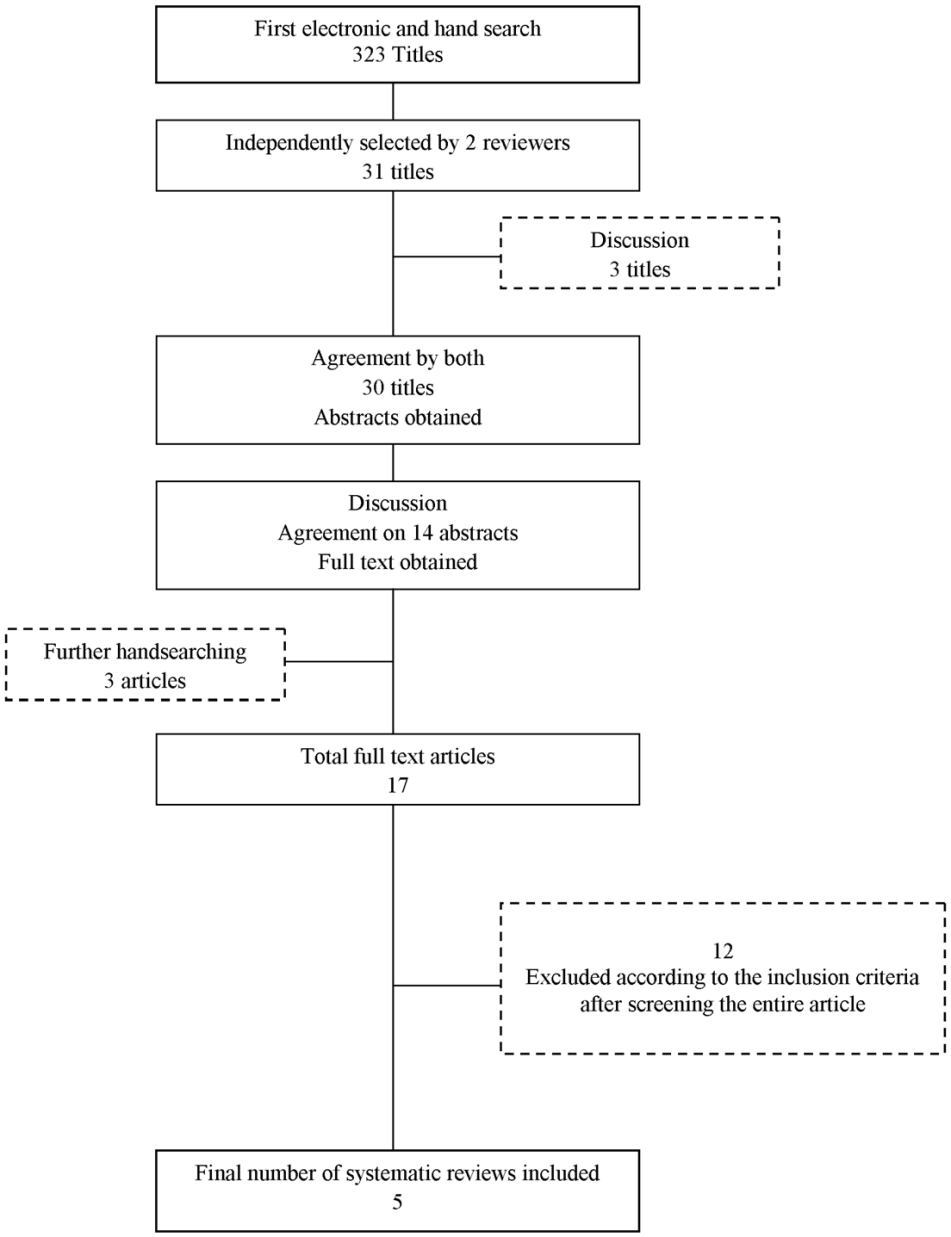
Flowchart of systematic review selection.

**Figure 2:**
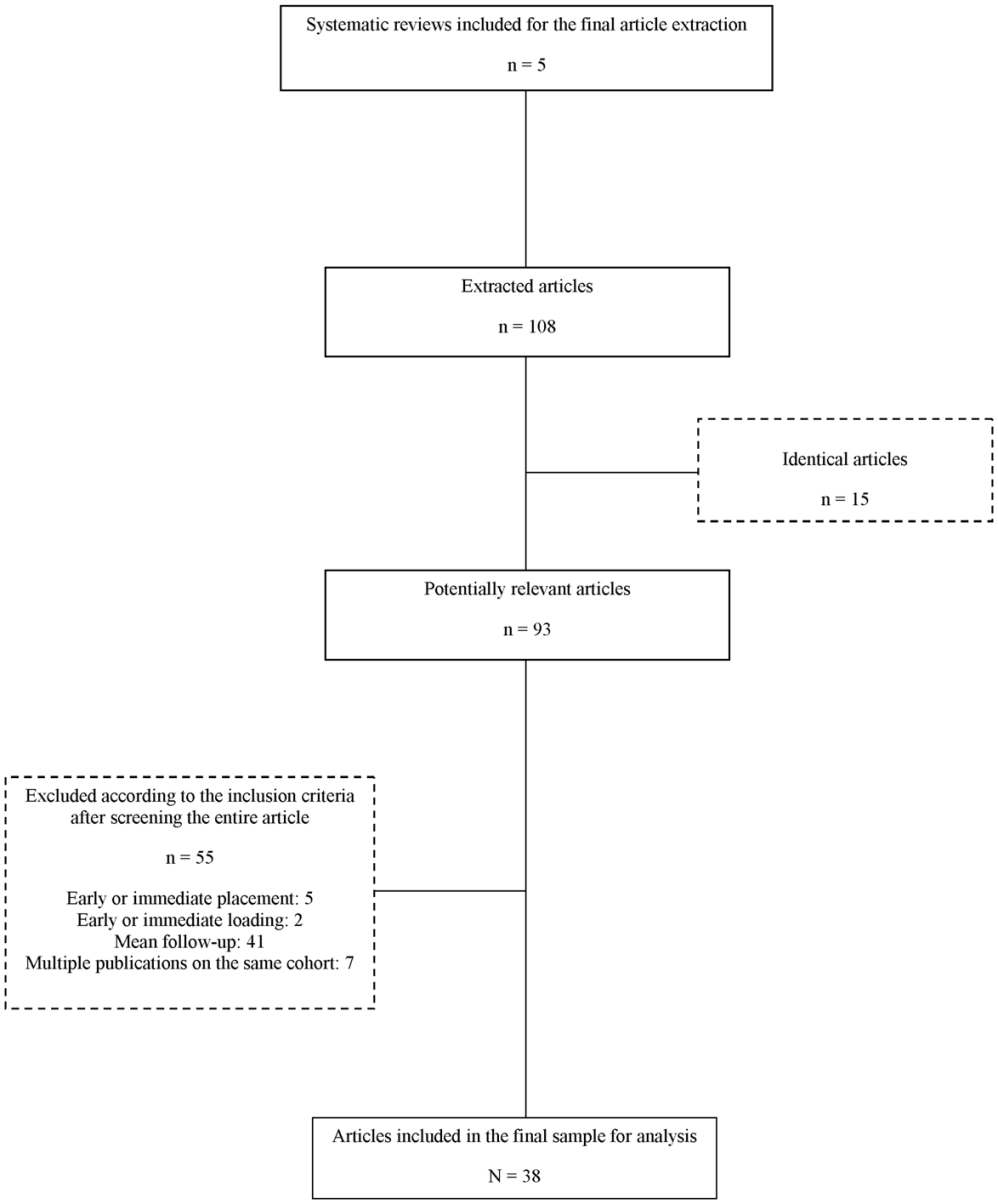
Flowchart of the primary article selection.

In the systematic review search, we screened a total of 323 articles and identified 17 articles of which, after full-text reading, 12 articles were excluded (Figure 1). Table S2 indicates the reason for exclusion of these articles. Therefore, a total of 5 systematic reviews were included to serve as the database from which primary articles were extracted [9], [10], [22], [29], [30]. Our final sample consisted of 38 published clinical trials. The full list of references which included primary articles is presented in Table S3. Figure 2 depicts the flow diagram for inclusion of the primary articles. Three articles dealt with 2 different prosthetic designs. They were thus each considered as 2 different trials for the analysis, leading to a total of 41 analyzable trials. The Kappa coefficient between examiners (A.P. and O.F.) was 0.90 (CI_95%_ [0.77–1.00]) demonstrating an almost prefect degree of agreement [31]. The initial Poisson model (with the offset variable only) provided a deviance equal to 161.20, which was much greater than the degrees of freedom (equal to 40), suggesting a strong heterogeneity.

Descriptive data for the extracted trials are shown in Table 1. The funding source was not reported in the majority of the trials (63%, 26/41). Taking into account all years together, the majority of the trials were considered as having a risk of bias (66%, 27/41). The reporting did not mention a statistical advisor in 30 trials (73%), and almost all trials did not report the periodontal status of the patients (83%, 34/41). Implant brands were dominated by one company (59%, 24/41), and Sweden, the company's original country, was the most common country among the corresponding authors (24%, 10/41). In our sample, 22% of the trials (9/41) were published in non indexed journals for the year prior to publication. A conflict of interest statement was disclosed in 2 trials. In both statements, the authors reported that they had no conflict of interest.

**Table 1:** Sample characteristics.

Characteristics	Category	Total n = 41
Age of publication, tertiles	4–10	31 (76%)
	11–15	9 (22%)
	>15	1 (2%)
Impact factors, tertiles	Not indexed	9 (22%)
	0.52–1.09	8 (19%)
	1.10–1.67	15 (37%)
	1.68–2.24	9 (22%)
Corresponding author country	USA	4 (10%)
	Belgium	5 (12%)
	Sweden	10 (24%)
	Switzerland	5 (12%)
	Italy	2 (5%)
	England	2 (5%)
	Germany	3 (7%)
	Canada	2 (5%)
	Others	8 (20%)
Study design	Retrospective	10 (25%)
	Prospective	31 (75%)
Quality assessment	Low risk of bias	14 (34%)
	Risk of bias	27 (66%)
Statistical advisor	Yes	11 (27%)
	No	30 (73%)
Prosthetic design	IS-SC	18 (44%)
	IS-FPD	15 (36%)
	ITS-FPD	8 (20%)
Periodontal status report	Yes	7 (17%)
	No	34 (83%)
Total number of implants, tertiles	10–347	38 (93%)
	348–685	2 (5%)
	686–1022	1(2%)
Number of failures, tertiles	0–19	37 (90%)
	20–39	2 (5%)
	40–58	2 (5%)
Implant brand	Straumann	8 (19%)
	Astra Tech	3 (7%)
	Nobel Biocare	24 (59%)
	Others	6 (15%)
Funding source	Industry	2 (5%)
	Industry associated	9 (22%)
	Non industry	4 (10%)
	Unknown	26 (63%)

**Figure 3:**
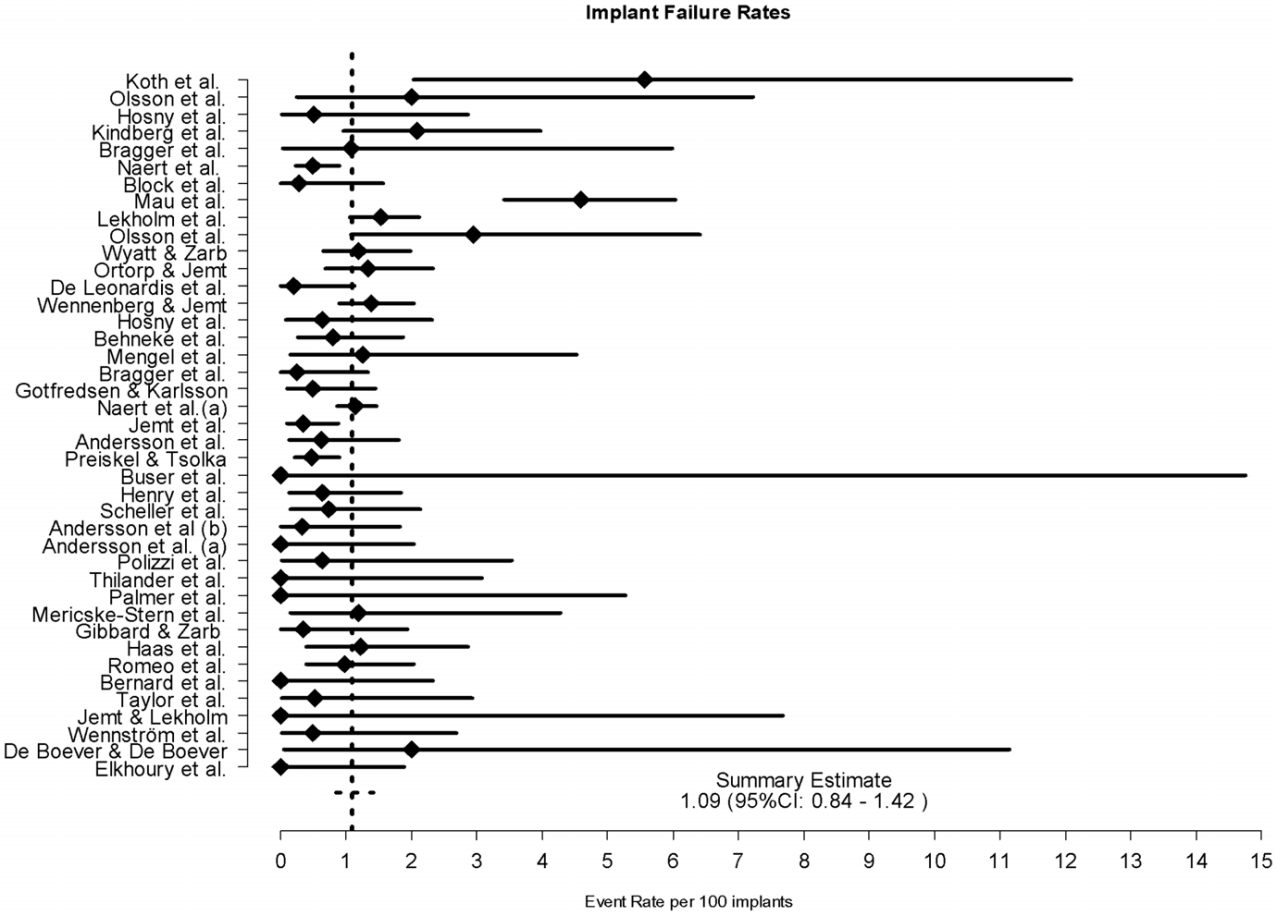
Annual percentages of failures.

Figure 3 indicates that the annual estimated percentages of failures ranged from 0 to 5.56 (CI_95%_ [0.00–14.76]). The mean annual failure rate of the trials was estimated at 1.09%.(CI_95%_ [0.84–1.42]). The mean annual failure rate calculated for the non-industry trials was equal to 2.73% (CI_95%_ [1.14–6.55], i.e. almost 3 times the rate for all the trials.

As shown in Table 2, the outcomes of the univariate quasi-Poisson regression models indicate a significant effect of the prosthetic design (p = 0.023) and the source of funding (p = 0.005). Compared with non-industry associated trials, both industry associated trials as well as trials where the sponsorship was unknown were more likely to report lower annual failure rates, showing ORs of 0.32 (CI_95%_ [0.17–0.60]) and 0.37 (CI_95%_ [0.21–0.63]), respectively. The multivariate analysis yields to the selection of publication age (p = 0.002) and funding source (p<0.001) in the final mode. Given data on funding source, the annual failure rate was 1.12 times higher for a one-year old increase in the publication age. Given information on study age, industry associated (OR = 0.21; CI_95%_ [0.12–0.38]) and unknown funding source (OR = 0.33; (CI_95%_ [0.21–0.51]) trials had lower annual failure rates as compared with non-industry associated trials.

**Table 2:** Regression quasi-Poisson univariate and multivariate models of the effects of independent variables on the annual failure rate of dental implants.

Category		Univariate Quasi-Poisson			Multivariate Quasi-Poisson		
		OR	CI 95%	p-value	OR	CI 95%	p-value
Study Age		1.07	[0.98–1.16]	0.085	1.12	[1.06–1.19]	0.002
Number of Implants		1.00	[0.99–1.00]	0.424			
Impact Factor		0.90	[0.61–1.33]	0.549			
Periodontal Status Report	No	1	–				
	Yes	0.90	[0.33–2.45]	0.812			
Prosthetic Design	ITS-FPD	1	–				
	IS-FPD	0.56	[0.34–0.91]				
	IS-SC	0.34	[0.15–0.77]	0.023			
Quality Score	LRB	1	–				
	RB	0.81	[0.47–1.41]	0.427			
Statistical Advisor	No	1	–				
	Yes	1.54	[0.95–2.50]	0.082			
Funding Source	Non-Industry Associated	1	–		1		
	Industry Associated	0.32	[0.17–0.60]		0.21	[0.12–0.38]	
	Unknown	0.37	[0.21–0.63]	0.005	0.33	[0.21–0.51]	<0.001

## Discussion

The main outcome of this study indicates that the funding sources have a significant effect on the annual rate of failure of dental implants. Given the publication age and when controlling for other factors, the annual failure rate for industry associated trials is significantly lower as compared with non-industry associated trials (OR = 0.21). These findings add significant new information to the field of dentistry and contribute to the body of literature showing that industry sponsoring may affect biomedical research outcomes [16], [32].

Interestingly, the multivariate analysis shows that trials where the financial funding source was not reported had an even lower probability of failure than industry associated trials (OR = 0.33). This outcome is hard to interpret. The trials belonging to this “unknown” category are probably the most biased. The authors deliberately did not report the sponsor. It may indicate that industry involvement in a clinical study implies some quality control to reduce bias in order to maintain company reputation. On the other hand, it is noteworthy that 63% of the trials included in the analysis did not report the funding source. This imbalance between sample size of disclosed and undisclosed funding sources (15/26) may have influenced the statistical results. The multivariate analysis also indicates that the annual rate of failure increases with the article seniority following the date of publication. This outcome may correspond to an improvement in dental implant therapies.

Leaving aside the lack of transparency of the funding source, a number of common weaknesses were identified in the trials'reporting. Lack of inclusion/exclusion criteria, inadequate blinding, and lack of drop-out rates were common, leading to the classification of 66% of the trials as having a risk of bias using our methodological quality assessment scale. Our intent was not to evaluate the overall quality of the trial, which may imply the rating of each individual feature aimed to reduce bias, but rather to provide a snapshot of the risk of bias of each trial through three items that focus on important aspects of study design. A statistician was identified in only 27% of the trials, rendering the analysis of the data questionable. In addition, the periodontal status of the subjects was reported in only 17% of the trials, although a significant difference in mean peri-implant marginal bone loss between patients with a history of periodontitis and periodontally healthy subjects has been shown [33], [34]. These weaknesses suggest that the relevance of the annual failure rates given by these types of trials must be interpreted with caution. However, taken together, these factors could not significantly overcome the impact of the funding source on the annual failure rate when included in the multivariate analysis.

We used the impact factor as the main journal characteristic because the quality of the reporting may vary with the journal. In our sample, only peer-reviewed journals were included. The regression models did not disclose an association between journal impact factors and the annual rate of failure. This finding is similar to the outcomes reported by other studies dealing with the sponsorship bias in pharmaceutical trials [16].

Our study is the first, to our knowledge, investigating the impact of sponsorship in the field of dentistry. This study has several strengths. We used dental implant therapy, a model where the market is very competitive as highlighted by the number of implant brands included in the sample. Thus, our findings may be generalizable to other dental products and devices within competitive markets, such as in orthodontics, periodontology or prosthetic dentistry. In addition, the consequences of the widespread use of dental implants have an effect on the health and well-being of the entire population, due to the impact on the extraction indications of the practitioners and on the health-policy aiming to reimburse dental therapies. Our study used the framework of a systematic method to identify the trials, and the annual failure rate as an objective measurable comparator. We decided to use the survival rate as the main variable and not the success rate because biological and technical complications were independently recorded in some systematic reviews [7], [8]. The overlap of biological and technical complications, due to the fact that one implant may have more than one complication, precludes any pooled estimate of success rate. Consequently, the number of systematic reviews using the survival rate as the main variable [9], [10], [22], [29], [30] was higher than those available using the success rate [30], [35].

The primary limitation of our study is the weak transparency of the financial support reporting of the trials included in the analysis. It may be assumed that among the 26 trials where the sponsorship was categorized as “unknown”, part of them were in fact partially supported by industry. For example, it is well-known that companies sometimes provide dental implants for free in order to evaluate their products. This may lead to publications and financial support of the companies in a communication plan where the authors are involved.

This “ghost” funding source may affect reporting objectivity. In addition, it was not possible to explore the relationship between a potential sponsor and the authors of the articles, because a conflict of interest statement was mentioned in only 2 out 41 trials. Consequently, it may be assumed that our study underestimated the number of industry associated trials.

Second, our sample is not exhaustive of all the studies dealing with dental implant survival. Systematic reviews were our source of trials because they are presumed to be objective. When done well, they are considered the highest level of evidence for medical decision making [36]. The results and conclusions of systematic reviews are often cited, and are the cornerstones of the decision making process when determining whether or not to insert an implant for tooth replacement. Thus, one can assume that the articles included in these reviews are those responding the best to high quality standards. Interestingly, in our study, 2 out of the 5 selected systematic reviews did not disclose their funding source [10], [29]. In the remaining 3 included systematic reviews, 2 of them declared no conflict of interest [9], [22].

Third, we were unable to pool meaningful evidences about factors that might be associated with the rate of failure. We thus speculated on variables identifiable in the sample assuming that they could affect the annual rate and be competitive with the sponsorship bias. Among them, the prosthetic design was regularly reported, and we hypothesized that it could be one of the most influential cofactors. We thus excluded trials involving fully edentulous patients in attempt to reduce the heterogeneity of the prosthetic design. Our hypothesis turned out to be strengthened by the significant effect of this variable in the univariate analysis (p = 0.023). However, the prosthetic design was not significant in the multivariate models, meaning that, even if strong, this factor could not overcome the funding source variable. Based on the basic principle that the magnitude of the treatment effect is influenced by the sample size, we also speculated that the sample size, i.e. the number of implants per trial, may have an effect on the rate of failure. Indeed, this variable did not show a significant effect in the present analysis.

Last but not least, all the prospective or retrospective trials that were included in the present analysis had an observational design and not an experimental design. It is well known that observational studies are more vulnerable to methodological problems and must be critically interpreted. They are prone to bias and confounding. Therefore, the real world mean annual failure rate is if anything likely higher than that found here for the non-industry trials (2.73%), given that the study designs have a high risk of bias.

The outcomes of this study strongly suggest the need for more transparency in the sponsorship of trials dealing with dental implant therapy. In the future, details on the financial source should be clearly reported. Dental publications should include at least a conflict of interest statement. So far, only one of the journals quoted in the present study has recently adopted such a statement. This is a first step towards increasing the transparency of the trial funding sources. Our results fully encourage editors of dental journals and authors of future studies in dentistry to be informed of certain initiatives that have been proposed to reduce the sponsorship bias in the biomedical research as well as in the Health Economics studies [15], [37]. Ultimately, more studies, including well conducted randomized clinical trials, are needed to identify potentially influential factors that can affect the rate of failure of the dental implants. Efforts should be made to increase the methodological quality of the reporting. So far, only 2 out of 9 dental journals involved in our sample (Journal of Clinical Periodontology and Clinical Oral Implant Research) have adopted the CONSORT guidelines [38]. The conclusions of the present study therefore strongly encourage the implementation of the CONSORT statements in the field of dental clinical research.

## Supporting Information

Table S1 MEDLINE (Pubmed) search strategy for systematic reviews selection.
(0.01 MB RTF)

Table S2 List of excluded systematic review articles and the reason for exclusion.
(0.05 MB RTF)

Table S3 List of included articles.
(0.07 MB DOC)
