# Supplementary material for: Relationship between Sponsorship and Failure Rate of Dental Implants: A Systematic Approach
Source: PLoS One. 2010 Apr 21;5(4):e10274. doi: 10.1371/journal.pone.0010274 (PMC2858083; doi:10.1371/journal.pone.0010274)
Supplement: Table S1 — MEDLINE (Pubmed) search strategy for systematic reviews selection. (0.01 MB RTF) [file pone.0010274.s001.rtf]

Table S1. MEDLINE (Pubmed) search strategy for systematic reviews selection

Limits Activated: Humans, Meta-Analysis, Review, English, Dental Journals, Publication Date: 1993-2008
#1 "Dental Implants"[Mesh] AND survival
#2 "Dental Implants"[Mesh] AND success
#3 "Dental Implants"[Mesh] AND complications 
#4 "Denture, Partial, Fixed"[Mesh] AND survival
#5 "Denture, Partial, Fixed"[Mesh] AND success
#6 "Denture, Partial, Fixed"[Mesh] AND complications
#7 (#1 OR #2 OR #3 OR #4 OR #5 OR #6): 323 articles
