# Supplementary material for: Relationship between Sponsorship and Failure Rate of Dental Implants: A Systematic Approach
Source: PLoS One. 2010 Apr 21;5(4):e10274. doi: 10.1371/journal.pone.0010274 (PMC2858083; doi:10.1371/journal.pone.0010274)
Supplement: Table S2 — List of excluded systematic review articles and the reason for exclusion. (0.05 MB RTF) [file pone.0010274.s002.rtf]

Table S2. List of excluded systematic review articles and the reason for exclusion

1.	Creugers NH, Kreulen CM, Snoek PA, de Kanter RJ (2000) A systematic review of single-tooth restorations supported by implants. J Dent 28: 209-217.    
Exclusion criteria: inadequate follow-up.   
                                                                                                                                                  
2.	Boioli LT, Penaud J, Miller NA (2001) meta-analytic, quantitative assessment of osseointegration establishment and evolution of submerged and nonsubmerged endosseous titanium oral implants. Clin Oral Implants Res 12: 579–588.
Exclusion criteria:  implant-supported SCs not reported.                                                                                                                                                   
3.	Berglundh T, Persson L, Klinge B (2002) A systematic review of the incidence of biological and technical complications in implant dentistry reported in prospective longitudinal studies of at least 5 years. J Clin Periodontol 29 Suppl.3: 197-212. 
Exclusion criteria: no data on implant survival rate
4.	Creugers NH, Kreulen CM (2003) Systematic review of 10 years of systematic reviews in prosthodontics. Intern J Prost 16: 123-127.    
Exclusion criteria: systematic review comparison.                                                                                                                                                                                                                                     
5.	Goodacre CJ, Bernal G, Rungcharassaeng K, Kan JY (2003) Clinical complications with implants and implant prostheses.  J Prosthet Dent 90: 121-132.
     Exclusion criteria: no data on implant survival rate.

6.	Schou S, Holmstrup P, Worthington HV, Esposito M (2006) Outcome of implant therapy in patients with previous tooth loss due to periodontitis.  Clin Oral Implants Res 17: 104–123.        
Exclusion criteria: no pooled survival rate reported.      
                                                    
7.	Pjetursson BE, Brägger U, Lang NP, Zwahlen M (2007) Comparison of survival and complication rates of tooth supported fixed dental prostheses (FDPs) and implant supported FDPs and single crowns (SCs).  Clin Oral Implants Res 18: 97–113.       
     Exclusion criteria: no data on implant survival rate.                                                                                          
8.	 Salinas TJ, Eckert SE (2007) In patients requiring single-tooth replacement, what are the outcomes of implant- as compared to tooth-supported restorations? Intern J Oral Maxillo Impl 22:71-95.                                                                                     Exclusion criteria: no data on implant survival rate.

9.	Ong CTT, Ivanovski S, Needleman IG, Retzepi M, Moles DR et al. (2008) Systematic review of implant outcomes in treated periodontitis subjects. J  Clin  Periodontol 35: 438–462.  
     Exclusion criteria: no pooled survival rate reported.                                                          
                                                                                                                                                                     
10.	Pjetursson BE, Lang NP (2008) Prosthetic treatment planning on the basis of scientific evidence. J  Oral Rehab 35: 72–79.         
Exclusion criteria: multiple publications on the same patient cohorts.    
                                                                                                                                  
11.	Tomasi C, Wennström JL, Berglundh T (2008) Longevity of teeth and implants – a systematic review. J Oral Rehab 35: 23–32.            
Exclusion criteria: no pooled survival rate reported.                                                                                                                                                                                    
12.	Torabinejad M, Anderson P, Bader J, Brown LJ, Chen LH et al. (2007) Outcomes of root canal treatment and restoration, implant-supported single crowns, fixed partial dentures, and extraction without replacement: a systematic review. J Prost Dent 98: 285-311.                                                                                                             Exclusion criteria: multiple publications on the same patient cohorts.                                                                                                                                      
                                    
