# Supplementary material for: Relationship between Sponsorship and Failure Rate of Dental Implants: A Systematic Approach
Source: PLoS One. 2010 Apr 21;5(4):e10274. doi: 10.1371/journal.pone.0010274 (PMC2858083; doi:10.1371/journal.pone.0010274)
Supplement: Table S3 — List of included articles. (0.07 MB DOC) [file pone.0010274.s003.doc]

List of included articles

1. Andersson B, Odman P, Lindvall AM, Branemark PI (1998) Cemented single crowns on osseointegrated implants after 5 years: results from a prospective study on CeraOne. Int J Prosthodont 11: 212–218.

*Funding source: Industry-associated*

1. Andersson B, Odman P, Lindvall AM, Branemark PI (1998) Five-year prospective study of prosthodontic and surgical single-tooth implant treatment in general practices and at a specialist clinic. Int J Prosthodont 11: 351–355.

*Funding source: Industry-associated*

1. Andersson B, Glauser R, Maglione, M, Taylor A (2003) Ceramic implant abutments for shortspan FPDs: a prospective 5-year multicenter study. Int J Prosthodont 16: 640–646.

*Funding source: Industry-associated*

1. Behneke A, Behneke N, d’Hoedt B (2000) The longitudinal clinical effevtiveness of ITI solid screw implants in partially edentulous patients: a 5year follow-up report. Int J Oral Maxillofac Implants 15: 633–645.

*Funding source: Unknown*

1. Bernard JP, Schatz JP, Christou P, Belser U, Kiliaridis S (2004) Long-term vertical changes of the anterior maxillary teeth adjacent to single implants in young and mature adults. A retrospective study. J Clin Periodontol 31: 1024–1028. *Funding source: Unknown*
2. Block MS, Lirette D, Gardiner D, Li L, Finger IM et al. (2002) Prospective evaluation of implants connected to teeth. Int J Oral Maxillofac Implants 17: 473–487.

*Funding source: Non-industry*

1. Brägger U, Karoussis I, Person R, Pjetursson BE, Salvi G et al. (2004) Technical and biological complications and failures with single crowns and fixed partial dentures on implant of the ITIs Dental Implant System: a 10-year prospective cohort syudy. Clin Oral Implants Res 16: 326-334 *Funding source: Non-industry*
2. Buser D, Dula K, Lang NP, Nyman S (1996) Long-term stability of osseointegrated implants in bone regenerated with the membrane technique. 5-year results of a prospective study with 12 implants. Clin Oral Implants Res 7: 175–183. *Funding source: Unknown*
3. De Boever AL, De Boever JA (2005) Guided bone regeneration around non-submerged implants in narrow alveolar ridges: a prospective long-term clinical study. Clin Oral Implants Res 16: 549–556.  *Funding source: Unknown*
4. De Leonardis DD, Gark AK, Pecora GE (1999) Osseointegration of rough acid-etched titanium implants: 5-year follow-up of 100 minimatic implants. Int J Oral Maxillofac Implants 14: 384–391.  *Funding source: Unknown*
5. Elkhoury JS, McGlumphy EA, Tatakis DN, Beck FM (2005) Clinical parameters associated with success and failure of single-tooth titanium plasma-sprayed cylindric implants under stricter criteria: a 5-year retrospective study. Int J Oral Maxillofac Implants 20: 687–694. *Funding source: Unknown*
6. Gibbard LL, Zarb G (2002) A 5-year prospective study of implant-supported single-tooth replacements. J Can Dent Assoc 68: 110–116.  *Funding source: Industry-associated*
7. Gotfredsen K, Karlsson U (2001) A Prospective 5-year study of fixed partial prostheses supported by implants with machined and TiO2-blasted surface. J Prosthodont 10: 2–7. *Funding source: Industry-associated*
8. Haas R, Polak C, Furhauser R, Mailath-Pokorny G, Dortbudak O et al. (2002) A long-term follow-up of 76 Branemark single tooth implants. Clin Oral Implants Res 13: 38–43. *Funding source: Unknown*
9. Henry PJ, Laney WR, Jemt T, Harris D, Krogh PH et al. (1996) Osseointegrated implants for single-tooth replacement: a prospective 5-year multicenter study. Int J Oral Maxillofac Implants 11: 450–455. *Funding source: Industry-associated*
10. Hosny M, Duyck J, van Steenberghe D, Naert I (2000) Within-subject comparison between connected and nonconnected tooth-to-implant fixed partial prostheses: up to 14-year follow-up study. Int J Prosthodont 13: 340–346. *Funding source: Unknown*
11. Jemt T, Bergendal B, Arvidson K, Bergendal T, Karlsson LD et al. (2003) Implant-supported welded titanium frameworks in the edentulous maxilla: a 5-year prospective multicenter study. Int J Prosthodont 16: 415–421. *Funding source: Unknown*
12. Jemt T, Lekholm U (2005) Single implants and buccal bone grafts in the anterior maxilla: measurements of buccal crestal contours in a 6-year prospective clinical study. Clin Implant Dent Relat Res 7: 127–135. *Funding source: Unknown*
13. Kindberg H, Gunne J, Kronström M (2001) Tooth- and implant-supported prostheses: a retrospective clinical follow-up up to 8 years. Int J Prosthodont 14: 575–581. *Funding source: Unknown*
14. Koth DL, McKinney RV, Steflik DE, Davis QB (1988) Clinical and statistical analyses of human clinical trials with the single crystal aluminium oxide endosteal dental implant: five-year results. J Prosthet Dent 60: 226–234. *Funding source: Unknown*
15. Lekholm U, van Steenberghe D, Herrmann I,Bolender C, Folmer T et al. (1994) Osseointegrated implants in the treatment of partially edentulous jaws: a prosopective 10-year multicenter study. Int J Oral Maxillofac Implants 9: 627–635. *Funding source: Industry-associated*
16. Mau J, Behneke A, Behneke N, Fritzmeier CU, Gomez-Roman G et al.(2002) Randomized multicenter comparison of two coatings of intramobile cylinder implants in 313 partially edentulous mandibles followed up for 5 years. Clin Oral Implants Res 13: 477–487. *Funding source: Non-industry*
17. Mengel R, Schröder T, Flores-de-Jacoby L (2001) Osseointergrated implants in patients treated for generalized chronic periodontitis and generalized aggressive periodontitis: 3- and 5-year results of a prospective long-term study. J Periodontol 72: 977–989. *Funding source: Unknown*
18. Mericske-Stern R, Grutter L, Rosch R, Mericske E (2001) Clinical evaluation and prosthetic complications of single tooth replacements by non-submerged implants. Clin Oral Implants Res 12: 309–318. *Funding source: Unknown*
19. Naert IE, Duyck JA, Hosny MMF, van Steenberghe D (2001) Freestanding and tooth–implant connected prostheses in the treatment of partially edentulous patients part I: an up to 15 years clinical evaluation. Clin Oral Implants Res 12: 237–244. *Funding source: Unknown*
20. Naert I, Koutsikakis G, Duyck J, Quirynen M, Jacobs R et al. (2002) Biologic outcome of implant-supported restorations in the treatment of partial edentulism. Part I: a longitudinal clinical evaluation. Clin Oral Implants Res 13: 381–389. *Funding source: Unknown*
21. Olsson M, Gunne J, Astrand P, Borg K (1995) Bridges supported by free-standing implants versus bridges supported by tooth and implant. A five-year prospective study. Clin Oral Implants Res 6: 114–121. *Funding source: Unknown*
22. Örtorp A, Jemt T (1999) Clinical experiences of implant-supported prostheses with laser-welded titanium frameworks in the partially edentulous jaw: a 5-year follow-up study. Clin Implant Dent Relat Res 1:84–91. *Funding source: Unknown*
23. Palmer RM, Palmer PJ, Smith BJ (2000) A 5-year prospective study of Astra single tooth implants. Clin Oral Implants Res 11: 179–182. *Funding source: Unknown*
24. Polizzi G, Fabbro S, Furri M, Herrmann I, Squarzoni S (1999) Clinical application of narrow Branemark System implants for single-tooth restorations. Int J Oral Maxillofac Implants 14: 496–503. *Funding source: Industry*
25. Preiskel HW, Tsolka P (2004) Cement- and screw-retained implant-supported prostheses: up to 10 years of follow-up of a new design. Int J Oral Maxillofac Implants 19: 87–91. *Funding source: Industry-associated*
26. Romeo, E, Lops D, Margutti E, Ghisolfi M, Chiapasco M et al. (2004) Long-term survival and success of oral implants in the treatment of full and partial arches: a seven-year prospective study with the ITI dental implant system. Int J Oral Maxillofac Implants 19: 247–259. *Funding source: Unknown*
27. Scheller H, Urgell JP, Kultje C, Klineberg I, Goldberg PV et al. (1998) A 5-year multicenter study on implantsupported single crown restorations. Int J Oral Maxillofac Implants 13: 212–218. *Funding source: Unknown*
28. Taylor RC, McGlumphy EA, Tatakis DN, Beck FM (2004) Radiographic and clinical evaluation of single-tooth Biolok implants: a 5- year study. Int J Oral Maxillofac Implants 19: 849–854. *Funding source: Unknown*
29. Thilander B, Odman J, Jemt T (1999) Single implants in the upper incisor region and their relationship to the adjacent teeth. An 8-year follow-up study. Clin Oral Implants Res 10: 346–355. *Funding source: Unknown*
30. Wennerberg A, Jemt T (1999) Complications in partially edentulous implant patients: a 5-year retrospective follow-up study of 133 patients supplied with unilateral maxillary prosteses. Clin Implant Dent Relat Res1: 49–56. *Funding source: Unknown*
31. Wennström, JL, Ekestubbe A, Grondahl K, Karlsson S, Lindhe J (2005) Implant-supported single-tooth restorations: a 5-year prospective study. J Clin Periodontol 32: 567–574. *Funding source: Industry*
32. Wyatt CCL, Zarb GA (1998) Treatment outcomes of patients with implant-supported fixed partial prostheses. Int J Oral Maxillofac Implants 13: 204–211.  *Funding source: Industry-associated*
